# Chromosomal loci important for cotyledon opening under UV-B in Arabidopsis thaliana

**DOI:** 10.1186/1471-2229-10-112

**Published:** 2010-06-16

**Authors:** Mariana Conte, Silvia de Simone, Susan J Simmons, Carlos L Ballaré, Ann E Stapleton

**Affiliations:** 1 Instituto de Investigaciones Fisiológicas y Ecológicas Vinculadas a la Agricultura, Consejo Nacional de Investigaciones Científicas y Técnicas and Universidad de Buenos Aires, C1417 DSE Buenos Aires, Argentina; 2 Department of Mathematics and Statistics, University of North Carolina at Wilmington, Wilmington, NC 28403 USA; 3 Department of Biology and Marine Biology, University of North Carolina at Wilmington, Wilmington, NC 28403 USA

## Abstract

**Background:**

Understanding of the genetic architecture of plant UV-B responses allows extensive targeted testing of candidate genes or regions, along with combinations of those genes, for placement in metabolic or signal transduction pathways.

**Results:**

Composite interval mapping and single-marker analysis methods were used to identify significant loci for cotyledon opening under UV-B in four sets of recombinant inbred lines. In addition, loci important for canalization (stability) of cotyledon opening were detected in two mapping populations. One candidate locus contained the gene *HY5*. Mutant analysis demonstrated that HY5 was required for UV-B-specific cotyledon opening.

**Conclusions:**

Structured mapping populations provide key information on the degree of complexity in the genetic control of UV-B-induced cotyledon opening in Arabidopsis. The loci identified using quantitative trait analysis methods are useful for follow-up testing of candidate genes.

## Background

Higher plants have complex sensory mechanisms to detect changes in the light environment [1-5]. There is evidence from physiological experiments that low levels of UV-B radiation (280-315 nm) induce photomorphogenic responses in *Arabidopsis thaliana *[6,7] and other species [8,9]. Results from studies of UV-B-induced photomorphogenesis [6] and gene expression [10-12] point to the existence of a complex web of interactions involving phytochromes and cryptochromes as a part of the underlying perception, regulation and/or signaling system [5]. However, several responses induced by low-dose UV-B appear to be to some extent independent of the photoreceptors that mediate responses to visible radiation [6,7,11,13]. Cotyledon-opening under defined UV-B conditions is a key phenotype, as this morphological response is consistent with signalling through a photoreceptor [6]. Fluence-response curves and the independence of the cotyledon-opening response from DNA repair make this defined cotyledon-opening phenotype especially useful for genetic screens [6].

Screens for isolating mutants with altered sensitivity to UV-B in hypocotyls have been performed and led to the identification of *uli3*, a UV-B hyposensitive mutant [13], and *uvr8*, a mutant hypersensitive to UV-B [14,15]. Both mutants appear to be altered in UV-B-specific signaling pathways. Photomorphogenic mutant analyses implicate the b-ZIP transcriptional factor HY5 as a component in the UV-B regulation of the expression of selected genes [11], and the *COP1 *gene as a regulator of *HY5 *and additional gene expression under UV-B [16]. Extensive analysis of UVR8 indicates that nuclear localization of the protein is important in UV-B signal transduction [17] and that UVR8 and HY5 are part of the same signal transduction pathway for gene expression responses [18].

Genome scanning is another useful approach for the isolation of loci that encode components of UV-B perception and signalling mechanisms. Genetic variability in wild ecotypes of *A. thaliana *provides an opportunity to discover genetic regions controlling phenotypic differences [19,20]. Genetically complex or polygenic inheritance, quantitative measurement and the availability of DNA markers allow access to quantitative trait loci (QTL) [21,22]. QTL mapping studies delimit the chromosomal regions controlling quantitative traits, and in some cases allow the identification of new alleles or causal mutations [23-25]. In the Arabidopsis model system QTL analysis has been performed with potentially adaptive traits like salt tolerance [26], aluminium tolerance [27], and powdery mildew disease resistance [28], as well as with developmental or physiological traits such as seed size [29], leaf architecture [30], inflorescence development [31] and growth rate [32]. It is possible to compare genetic architecture in different environments, including controlled treatment environments, although the power to detect loci will be less than the detection power for overall genetic effects [33].

Metabolic and signalling pathways can be identified from epistatic interactions [34-37], and gene interactions are widespread and important in theory and in most data sets. Epistatic interactions can explain substantial amount of variance [38], although power to detect epistasis is low in most recombinant inbred (RI) experiments with 100-200 lines. Better understanding of the genetic architecture of UV-B responses will thus be achieved by incorporation of epistatic locus interactions into the analysis.

Differences in phenotype measurements between individuals of the same RI genotype are due to random fluctuation and alleles genetically controlling stability (canalization, for developmental traits) or variance (anti-canalization) [39,40]. Arabidopsis loci for canalization of flowering and plant growth have been found previously, by QTL mapping of RI lines [41]. Full understanding of phenotype and environmental constraints on phenotype expression require consideration of the stability of the phenotype as well as the extent of the effect of important alleles.

No QTL studies have been reported with UV-B-induced responses in Arabidopsis. In rice Sato et al. [42] have reported three QTLs controlling UV-B resistance, with a more precise mapping of the location of one of the reported QTLs [43]. Recombinant inbred lines, or in some species clones of F_2 _families, are the most useful for exploration of genotype and environment interactions, as the same genotype can be exposed to multiple environments [33].

In the experiments described in this paper we used four sets of RI lines to perform a QTL analysis of UV-B-induced photomorphogenesis in Arabidopsis. We measured cotyledon opening in de-etiolating seedlings as a model photomorphogenic response; under certain experimental conditions this response is induced by UV-B (but not UV-A) and the induction mechanism for cotyledon opening does not appear to involve signals derived from UV-B-induced DNA damage [6]. We used composite interval mapping to locate additive, UV-B-specific and epistatic QTL, by incorporating all the data including replicates and multiple 'environments', which in this case are UV-B treatment and control (-UV-B) growth conditions. We also analyzed these data with a straightforward single-marker method to select the most robust loci for candidate gene hypothesis testing. Loci for cotyledon-opening stability were identified; these include loci at the same position as the cotyledon opening QTL and new loci that regulate stability but not extent of cotyledon opening. We combined our QTL with information from the literature to produce a list of candidate genes for further testing. Mutations in our top candidate gene, *HY5*, were defective in UV-B-induced cotyledon opening.

## Results and Discussion

**Figure 1:**
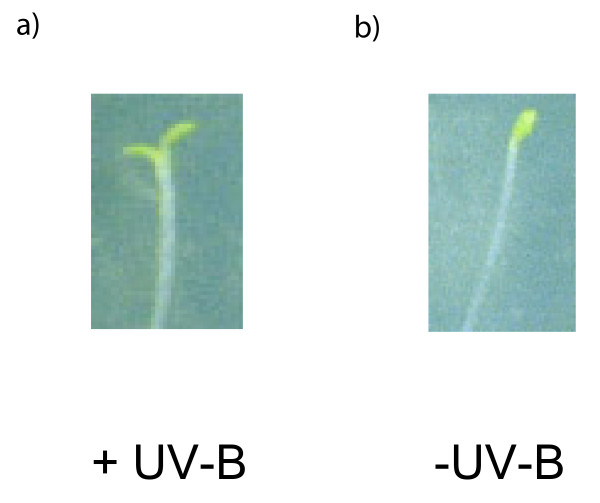
**UV-B-induced Cotyledon Opening**. Wildtype Columbia seedlings were grown and irradiated as described in Materials and Methods. a) +UV-B (cellulose di-acetate) treatment b) -UV-B (Mylar) control.

Cotyledon opening in Arabidopsis was induced specifically by low levels of UV-B, and not by control irradiations without UV-B, as illustrated in Fig. 1. Cotyledon-opening angle is a UV-B-photoreceptor-induced response [6], and was thus the phenotype used for our mapping experiments. The UV-B treatment was given to young dark-grown seedlings from the RI populations as a 2.5 hr low-fluence pulse, as previously described for this UV-B-cotyledon-opening response [6]. Statistical analysis of these +UV-B and -UV-B measurements allowed identification of a total of 21 loci, with 16 loci controlling cotyledon opening (14 with additive main effects and two that were epistatic-only), four loci controlling UV-B-specific cotyledon opening, and three loci controlling the stability of cotyledon-opening.

### Chromosomal regions affecting cotyledon opening

**Figure 2:**
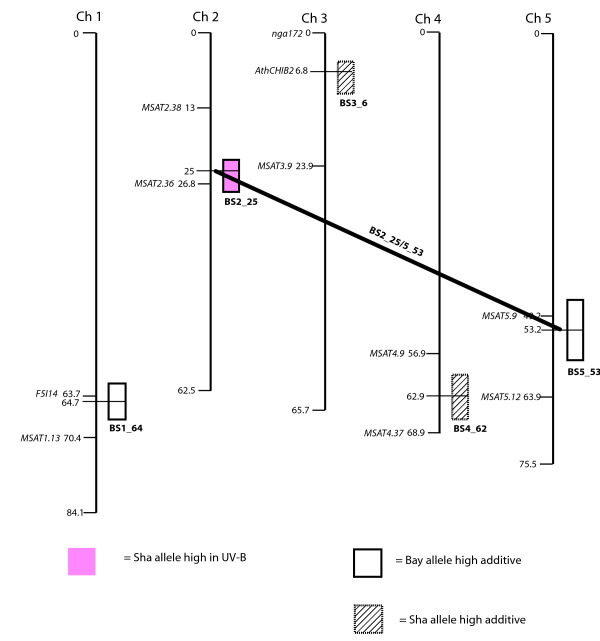
**Genomic regions with significant trait associations in the Bay X Sha recombinant inbred population**. Colored boxes indicate regions associated with UV-B-induced cotyledon opening (under cellulose di-acetate). White and hatched boxes indicate regions associated with cotyledon opening with no significant environmental effect in the comparison of UV-B treatments with control no-UV-B (Mylar) treatments. Colored boxes indicate UV-B-specific loci. Horizontal lines connect epistatic QTL. Significant loci are named by the population, chromosome, and map distance.

**Figure 3:**
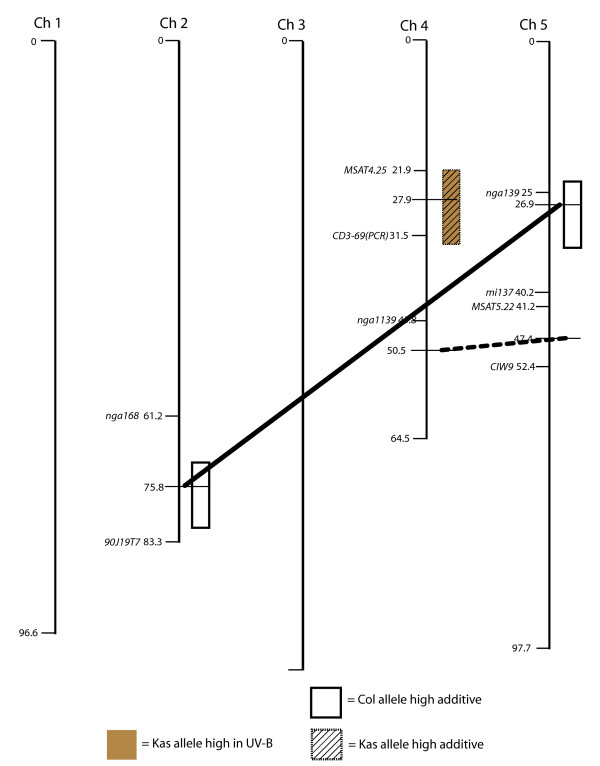
**Genomic regions with significant trait associations in the Col X Kas recombinant inbred population**. Colored boxes indicate regions associated with UV-B-induced cotyledon opening (under cellulose di-acetate). White and hatched boxes indicate regions associated with cotyledon opening with no significant environmental effect in the comparison of UV-B treatments with control no-UV-B (Mylar) treatments. Colored boxes indicate UV-B-specific loci. Horizontal lines connect epistatic QTL. Significant loci are named by the population, chromosome, and map distance.

**Figure 4:**
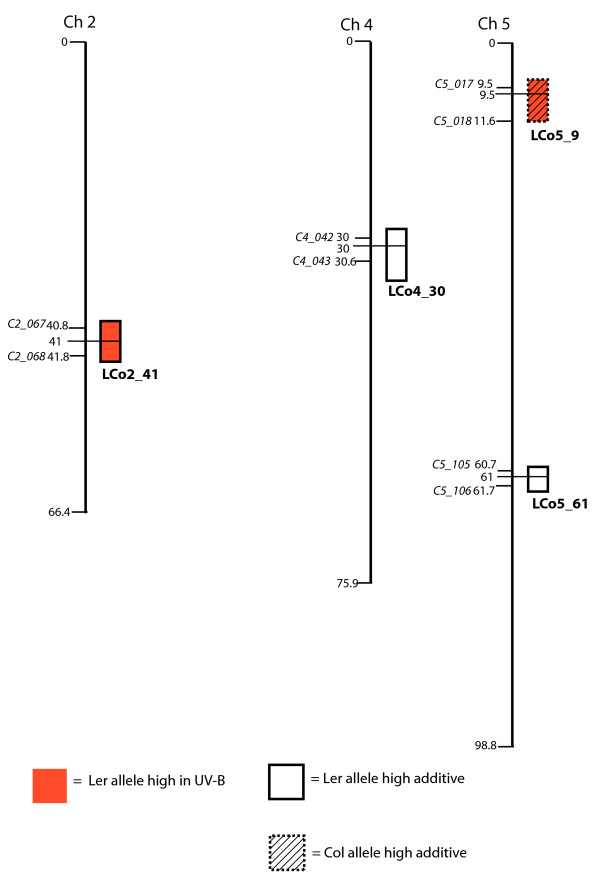
**Genomic regions with significant trait associations in the Ler X Col recombinant inbred population**. Colored boxes indicate regions associated with UV-B-induced cotyledon opening (under cellulose di-acetate). White and hatched boxes indicate regions associated with cotyledon opening with no significant environmental effect in the comparison of UV-B treatments with control no-UV-B (Mylar) treatments. Colored boxes indicate UV-B-specific loci. Horizontal lines connect epistatic QTL. Significant loci are named by the population, chromosome, and map distance.

**Figure 5:**
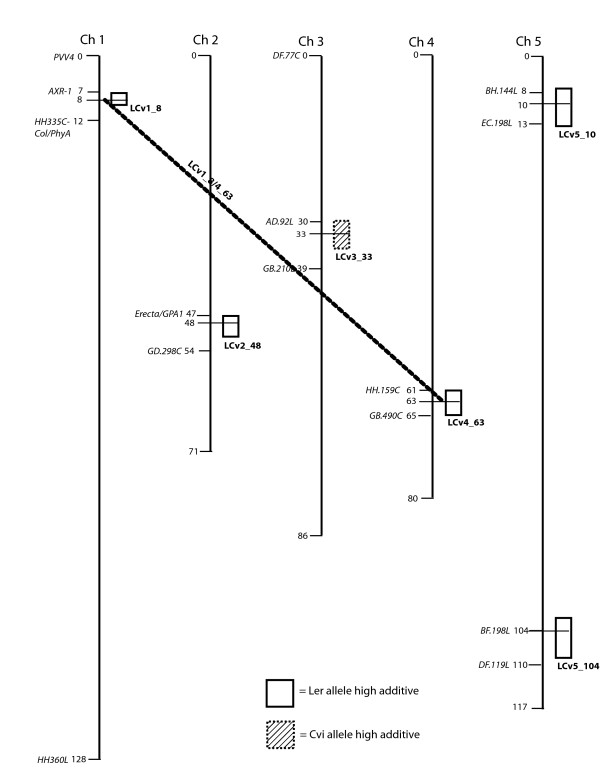
**Genomic regions with significant trait associations in the Ler x Cvi recombinant inbred population**. Colored boxes indicate regions associated with UV-B-induced cotyledon opening (under cellulose di-acetate). White and hatched boxes indicate regions associated with cotyledon opening with no significant environmental effect in the comparison of UV-B treatments with control no-UV-B (Mylar) treatments. Colored boxes indicate UV-B-specific loci. Horizontal lines connect epistatic QTL. Significant loci are named by the population, chromosome, and map distance.

A total of 16 separate loci that control cotyledon opening are significant at our stringent experiment-wise P < 0.05 threshold (Figs. 2, 3, 4, 5). Loci are identified by chromosome and map distance codes. Details of the analysis, including estimated effect sizes for contrasting alleles, are given in Additional File 1, Additional File 2, Additional File 3 and Additional File 4, arranged by locus identification code. Fifteen of these loci were also significant in the single-marker analysis. The amount of variance explained by difference in genotype differs in the four mapping populations, with LerxCol and LerxCvi having the highest heritabilities. Marker spacing is most dense in LerxCol and LerxCvi, and most sparse in BayxSha. The combination of heritability of the trait, partition of the genome into larger numbers of RI lines, and the marker parameter space determines the power to detect loci [33]; as expected, we detected the most loci in LerxCvi and BayxSha populations. New genotyping methods can locate all the recombination junctions in RI populations [44], although the specific lines in our study have not yet been genotyped to this density.

### Comparison of epistatic interactions

Gene interactions (epistasis) are likely to be important in explaining genetic control of phenotypic traits; however, these interactions are difficult to detect [45]. We scanned for interactions between two loci, whether or not those loci had an effect on cotyledon opening considered singly. Significant two-locus epistatic interactions were detected in the BayxSha (Fig. 2), ColxKas (Fig. 3) and LerxCvi (Fig. 5) populations. Larger mapping populations are needed for detection of multiple loci by interval mapping methods [33], and these are the larger mapping populations in our study. In BayxSha and LerxCvi the epistatic interactions had lower heritability than any individual locus, suggesting only small contributions to genetic control of cotyledon opening. In contrast, for the ColxKas population epistatic interactions are important, with heritabilities as high as those seen in individual main-effect loci (Additional File 2).

The BayxSha loci with significant epistasis also had significant main effects (Fig. 2), although the main effects were opposite in their allele contributions (Sha allele with the higher effect in BS2_25 and the Bay allele having the larger effect at BS5_53, Additional File 1). Thus, the epistatic interaction is not a simple additive effect. The estimated epistatic high allele is Bay, which suggests that the Bay allele at BS5_53 interacts equally well with either allele from the BS2_25 region, but the Sha allele at BS5_53 is more specific.

As in BayxSha, the LerxCvi epistatic loci also have single-locus main effects. In this case, both single-locus Ler alleles contribute to cotyledon opening while the epistatic alleles are from Cvi. This suggests that the Cvi alleles contribute to a pathway or complex, while the products of the Ler alleles work independently.

In the ColxKas population with the CK2_75/5_27 pair the allele effect was of the same direction as the individual locus alleles and of intermediate effect size. In this population there was also an epistatic interaction between two new loci on chromosomes 4 and 5; these new loci had no significant effect considered separately (Fig. 3). This is an example of epistasis with no main effect, which may indicate the epistatic alleles have a negative effect on their interaction partner.

Detection of interactions between loci may be useful for predicting which genes under the QTL regions are causal, as metabolic networks and gene families can cause epistasis [34-37]. The four mapping populations vary in extent of epistasis effects, indicating that particular allele interactions underlay the statistical epistasis we detected.

### Chromosome regions with a UV-B-specific effect

Four loci with significant effects in the +UV-B-exposed treatment environment (cellulose di-acetate filter) were identified, BS2_25 from the BayxSha population, CK3_28 from the ColxKas population, and LC2_43 plus LC5_8 from the LerxCol population (Figs. 2, 3, 4, 5). Significant loci detected explained only about one-fourth of the genotype by environment variance. The remaining variance could be explained by presence of a number of additional small-effect loci or by epistatic interactions among more than two markers in these populations.

### Architecture of canalization loci

The striking differences in the range of cotyledon opening in the RI lines (Additional File 5, Additional File 6, Additional File 7, Additional File 8, Additional File 9, Additional File 10, Additional File 11, Additional File 12) suggested that genes for the stability of the trait might be segregating in these mapping populations. Stability (canalization) is known to have a genetic component in flowering and general growth in the LerxCol and LerxCvi populations [41]. We found QTL for cotyledon-opening variance in three populations (Table 1). As expected from previous work, some QTL alleles confer both an effect on the amount of cotyledon opening and an effect on the variance. The cotyledon-opening stability QTL in LerxCol (S.LCo2_45) overlaps with the ERECTA region QTL found by Hall et al. [41]. Cotyledon-opening stability was also controlled by previously unidentified loci on Chromosome 4 in LerxCol and Chromosome 3 in BaySha (Table 1). The Chromosome 4 S.LCo4_57 locus spans 0.2 Mb; candidate genes in this interval include a protein disulfide isomerase (At4g27080) [46] and an ascorbic acid biosynthesis gene, VTC2 [47]. The vtc2 mutant appeared to have higher variance in vegetative growth in previous studies [48]. The S.LCo2_45 erecta-region stability QTL also has an allele-specific UV-B effect; the Ler allele variance is higher in the +UV-B environment.

**Table 1:** Genomic Loci for Canalization

ID	Chromosome	Marker Interval	QTL Position in cM	QTL Position Range in cM	High-Effect Allele	QTL Pvalue	**h**^**2 **^**of QTLs**	Comparison to cotyledon opening QTL
Bay x Sha population^1^								

S.BS3_10	*3*	ATHCHIB2-MSAT3.19	10.8	4 - 17.8	Bay	P = 6.9 × 10^-4^	0.016	Same locus; higher variation allele has lower effect on opening.

Ler x Col population^2^								

S.LCo2_45	*2*	C2_074-C2_075	45.6	41 - 54				Locus is the same or nearby (intervals overlap), has significant UV-B variation effect but no additive main effect. Ler allele median variance is high in +UV-B round 1 (P = 0.0039), and Ler allele median variance is low in -UV-B round 2 (P = 0.016).

S.Lco4_57	*4*	C4_089-C4_090	57.1	55 - 58	Col	P = 3.5 × 10^-5^	0.043	New locus, no UV-B-specific effect.

^1^BayxSha QTL variance components: Vg/Vp = 0.016 Ve/Vp = 0.1675 Vge/Vp = 0.014 Vr/Vp = 0.864*

^2^LerxCol QTL variance components: Vg/Vp = 0.05 Ve/Vp = 0.266 Vge/Vp = 0.0744 Vr/Vp = 0.6094*

*Vg is variance of genetic main effects, Vp is phenotypic variance, Ve is environmental (UV-B) effects, Vge is variance of genotype-by-environment interaction effects, Vr is residual variance.

### Comparisons of chromosomal regions detected in the different populations

We do expect to have the same alleles in geographically distinct accessions if there is strong selection. There is some overlap in the accession represented in the mapping populations, in that Ler is in two populations and Col in two populations. The Col common parent does not condition any common loci but in mapping populations with a Ler parent, a region in the top of Chromosome 5 is present. The additional support for these loci derived from detection in multiple populations illustrates the value of including common parents in mapping populations or using a diallel design to derive RI lines [49,50].

### Candidate genes

We examined chromosomal regions spanned by the four UV-B-specific QTL for genes known to be important in UV-B signalling (Table 2) and for genes with UV-affected expression profiles or annotations suggesting UV function (Table 3).

**Table 2:** Comparison of UV-B signaling gene position to QTL

UV-B signaling gene	comparison to QTLs	comments
Uvr8 [15,18]	No overlap	

Hy5 [11]	LCo5_9 UV-B specific QTL, also LCv5_10	

Hyh [18]	BS3_6 UV-B-specific QTL	Partially redundant with Hy5

Cop1 [16]	No overlap	Cop1 has pleiotrophic effects

Uli3 [13]	LCv5_104	

**Table 3:** Highest priority candidate genes under UV-B-specific QTL

QTL	candidate	comment
LCo5_9	HY5	also LCv5_10

BS2_25	At2g07190 hsp27-like	Large QTL region that includes CEN2

CK4_27	At4g31500 RED1	RED1 is downregulated by PHYB [67] and has auxin phenotypes.

LCo2_41	At2g26710 light signaling	Involved in multiple light signalling pathways; UV-regulated expression [11]

We selected criteria for searching for candidate genes under QTL by considering 1) loci found in multiple populations with common alleles 2) loci with UV-B-specific effects 3) loci confirmed with both QTL Network and single-marker methods 4) size of region, and 5) the availability of additional information such as expression data on genes located near significant markers and/or annotation of genes with suggestive biochemical functions [21]. Based on these criteria, a region on the top of Chromosome 5 was selected.

### Candidate gene mutant *hy5 *has a defect in UV-B-induced cotyledon opening

In the LerxCol and LerxCvi populations there is a region at the top of chromosome 5 identified by QTLNetwork and single-marker analysis as controlling cotyledon opening. Examination of this region of the AGI Arabidopsis map http://www.arabidopsis.org suggested one obvious candidate gene, *HY5*/At5g11260, in this interval. Global gene expression experiments have implicated the *HY5 *gene in signal transduction from a UV-B receptor [11] and additional expression measurements have placed the *UVR8 *and *HY5 *genes in that same signal transduction pathway [18].

We examined the cotyledon-opening angles by calculating the medians split on significant parent markers for Ler x Cvi and Ler x Col lines. For those Ler x Cvi lines that have the BH.144L Ler marker the UV-B opening angle is greater (median 101) than when Cvi allele is present (median 74); thus the Ler allele of this marker is responsive and Cvi allele is less responsive. For the Ler x Col population, marker C5_017 has a higher median for the Ler allele group than the Col allele subset, although the UV-B treatment difference is not significant in the QTL Network analysis. This suggests that the actual locus conditioned by the Ler responsive allele is between anchor markers nga249 and nga151 on the physical map; this region includes the *HY5 *gene. We examined the available Ler genomic sequence [51] in this chromosomal region. There were several polymorphisms in both the coding region and in the 5' UTR region of the *HY5 *gene.

**Figure 6:**
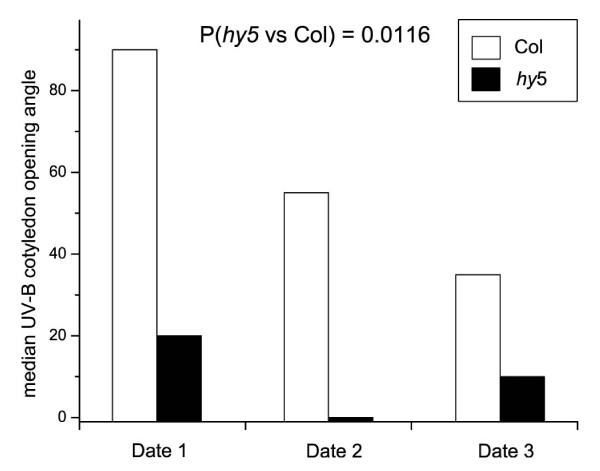
**UV-B-induced cotyledon opening in ecotype Columbia and *hy5-215***. The median cotyledon opening angle for each line in each of the three separate experiments is shown. Overall P value from a general linear model for the comparison of the extent of opening in the two lines is P = 0.0116. Interactions between experiment number and line were significant. For experiment 1, Col n = 15, *hy5 *n = 15. For experiment 2, Col n = 10, *hy5 *n = 16. For experiment 3, Col n = 10 and *hy5 *n = 10. Pairwise permutation tests on each time point individually gave P < 0.0001.

As Vreugdenhil et al. [52] note, the presence of multiple polymorphisms makes testing of mutants the most efficient strategy for QTL cloning in Arabidopsis. Thus, we examined UV-B-induced cotyledon opening in a *HY5 *mutant, *hy5-215*. The mutant was defective in UV-B-induced cotyledon opening (Fig. 6). This is the first identification of the role of HY5 in the low-fluence cotyledon-opening responses to UV-B, which suggests that the gene expression responses controlled by UVR8 and HY5 [18] are also involved in UV-induced morphological responses in de-etiolating seedlings. In the future candidate gene insertion mutations near the other UV-specific loci that we have identified could be tested to connect cotyledon opening phenotype differences to additional specific genes.

## Conclusions

Extensive characterization of morphological responses such as cotyledon-opening, when combined with genotyped structured mapping populations, allow identification of chromosomal loci and specific locus interactions important for control of the growth response. In addition to the region near *HY5*, we have identified several other loci associated with UV-B-induced cotyledon opening as well as loci specifying general light-induced opening.

## Methods

### Plant material

Four recombinant (RI) line sets of *A. thaliana *were used for QTL mapping of cotyledon opening induced by UV-B. Lines used in this work were obtained from the Arabidopsis Biological Resource Center (ABRC) in Columbus, Ohio http://www.arabidopsis.org. The accession numbers include CS57921 for Bay x Sha, with 165 RILs developed by [53], CS84999 for Col x Kas, with 128 RILs developed by [28], CS1899 for Ler x Col, with 99 RILs (one is redundant), developed by [54], and CS22000 for Ler x Cvi, with 162 RILs developed by [55]. The *hy5-215 *mutant seeds in the Columbia background were kindly provided by X.-W. Deng (Yale University).

### QTL experimental design

We designed our QTL-mapping experiment following the guidelines described by Belknap [56] and Lynch and Walsh [33], with the additional priority of ensuring that populations with multiple parent ecotypes were included [57]. As our priority was detection of loci important for UV-B-induced cotyledon opening, we chose to measure more replicates. This allows better estimation of effect sizes (amount of cotyledon opening conferred by the two alternative alleles at a locus), at the cost of less precise estimation of the position of the QTL on the map and less ability to detect 'small' QTL that have relatively little effect on the response [56,58]. Measurements on each RI line under each treatment (+UV-B and -UV-B) were repeated, with the two sets of measurements named round1 and round2 (Additional File 5, Additional File 6, Additional File 7, Additional File 8, Additional File 9, Additional File 10, Additional File 11, Additional File 12). Eight seeds were planted for each treatment-RI line combination for each round. The two rounds were analyzed separately, in a way similar to traditional crop QTL analyses where planting year is analyzed as a separate factor.

### Plant growth conditions

Eight seeds of each RI line or 16 seeds of *hy5-215 *and Col wildtype [59,60] were sown in 1 cm height plastic boxes containing 0.8% agar (w/v). Boxes were covered with UV-B transparent film (Rolopac, Buenos Aires, 0.025 mm) and stored for three days in darkness at 6°C. To induce germination, seeds were exposed to an R-light pulse (30 minutes) and incubated in darkness at 25°C for 24 hours before being transferred to the UV-B irradiation chambers. R light (30 μmoles m^-2 ^s^-1^) was provided by red fluorescent tubes (40/15, Philips).

### UV-B treatment

UV-B treatment and measurement of cotyledon opening were carried out as previously described [6]. Briefly, one-day-old etiolated seedlings were exposed for 3 days to a daily period of 2.5 hours of UV-B followed by a 5 minute R pulse. Seedlings were transferred to the dark for 24 hours and then the aperture of the cotyledons was measured on the fourth day. UV-B was provided by two UV-B 313 bulbs (Q-Panel 313, Cleveland), with a 0.1-mm-thick cellulose di-acetate film (La Casa del Celuloide, Buenos Aires) placed between the tubes and the seedlings to filter out the UV-C radiation emitted by the fluorescent tubes, as previously described [12,15]. UV-B level was measured with a IL1700 double-monochromator spectroradiometer (International Light, Newburyport, MA), integrating the spectral irradiance between 290 and 315 nm. The radiometer was calibrated against a standard lamp (OL-40, Optronic, Orlando, FL) in the short-wavelength range and a model 1800 calibrator (LI-COR, Lincoln, NE) for wavelengths greater than 320 nm. In order to obtain a control treatment (-UV-B treatment), the UV-B portion of the spectrum emitted by the Q-Panel 313 bulbs was removed using a clear polyester film (Mylar-D, DuPont, Wilmington, DE; 0.1-mm thick). Neutral density filters were placed below the filters to reduce the intensity of UV to 2.25 μmoles m^-2 ^sec ^-1^. This photon flux was identified as a suitably low level from the action spectrum for UV-B-induced-cotyledon-opening [6]. UV spectra under each film type are provided in Additional File 13.

### Measurement of cotyledon opening

Cotyledon aperture was measured with a protractor and magnifying glass. Accuracy of measurement for each assayer was checked by measuring test seedlings 6-9 times; the coefficient of variation of the measurements within and between raters was less than 5% (data not shown). Measurements were expressed as angle between the cotyledons (0 to 180°). The raw measurement data (in degrees) on all individual plants is available in Additional File 5, Additional File 6, Additional File 7, Additional File 8, Additional File 9, Additional File 10, Additional File 11 and Additional File 12, arranged by population and date of measurement.

### Molecular markers

These RI lines have been characterized for molecular markers and that data was accessed from the Natural resource at http://arabidopsis.info/BrowsePage. We removed uninformative markers from our analysis by checking each adjacent marker for difference in the line distribution pattern of the markers. If the line distribution pattern in two successive markers on the chromosome was exactly the same, the second marker was removed. In order to determine if there was any artificial (non-syntenic) correlation in the particular RI line sets that we used, we performed Pearson correlations on each line set, as previously described [61], using the SAS procedure CORR. There was little artificial correlation evident in the line sets we used (data not shown).

### Variance phenotype

To adjust for the large numbers of zeros in the data set, 0.5 was added to each phenotype measurement. Levene's median natural log statistic for each individual was then calculated as previously described [41,62]. Using the median form is a conservative choice as compared to use of the mean version of the Levene's statistics, as the median form is less sensitive and less susceptible to artefacts with increasing variance in larger trait measurements. The full data set was used for QTLNetwork analysis.

### QTL Network v. 2.0 analysis

This composite interval mapping program [63] was accessed from http://ibi.zju.edu.cn/software/qtlnetwork/. This particular CIM mapping program was chosen as it has provision for appropriate analysis of multiple replicates of each line and for multiple environments (+UV-B and -UV-B, in our case) [64]. This program estimates effect sizes using a Bayesian fitting method [64], and chooses cofactors automatically. Program default settings (test window of 10 cM, walk speed of 1 cM) were used, except for deselection of the option for best genotype prediction. The experiment-wise P value threshold (across the whole experiment, two rounds of two treatment environments for eight replicate genotypes of each RIL in each of the four populations) was kept at P = 0.05, with 1000 permutations for F-value threshold selection.

### Marker-based analysis

A mixed model was constructed in SAS v9.1 (SAS Inc, Cary, NC) for individual marker state and UV-B treatment. Each round of the experiment was analyzed separately. A genome-wide P value threshold of 10^-4 ^was chosen [33,65], as the marker density in these RI line sets was not large enough to require adjustment for marker correlation [66].

### *hy5 *data analysis

Cotyledon opening angles in mutant and Columbia wild-type were compared in individual experiments by permutation test and then in all three experiments. The modeling of all *hy5 *and Col measurements was performed using SAS PROC GENMOD with the angle measurements divided by ten to generate truncated values between 0 and 18 and specifying a Poisson distribution.

## Authors' contributions

This project was conceived by AS, who provided grant support for seed purchase and travel. MC and SdS grew the plants and measured cotyledon opening. SS and AS carried out the data analysis. MC, AS and SS wrote the manuscript; CB edited the manuscript, provided support for MC and SdS, and hosted AS in his lab. All authors read and approved the final manuscript.

## Supplementary Material

Additional File 1 **Details about significant quantitative trait loci from the BayxSha mapping population**. Click here for file

Additional File 2 **Details about significant quantitative trait loci from the ColxKas mapping population**. Click here for file

Additional File 3 **Details about significant quantitative trait loci from the LerxCol mapping population**. Click here for file

Additional File 4 **Details about significant quantitative trait loci from the LerxCvi mapping population**. Click here for file

Additional File 5 **All measured cotyledon-opening angles, arranged by experimental round and RIL population in excel spreadsheet format (xls).** Each file contains angles for with both +UV-B (cellulose di-acetate) and -UV-B (Mylar). Click here for file

Additional File 6 **All measured cotyledon-opening angles, arranged by experimental round and RIL population in excel spreadsheet format (xls).** Each file contains angles for with both +UV-B (cellulose di-acetate) and -UV-B (Mylar). Click here for file

Additional File 7 **All measured cotyledon-opening angles, arranged by experimental round and RIL population in excel spreadsheet format (xls).** Each file contains angles for with both +UV-B (cellulose di-acetate) and -UV-B (Mylar). Click here for file

Additional File 8 **All measured cotyledon-opening angles, arranged by experimental round and RIL population in excel spreadsheet format (xls). **Each file contains angles for with both +UV-B (cellulose di-acetate) and -UV-B (Mylar). Click here for file

Additional File 9 **All measured cotyledon-opening angles, arranged by experimental round and RIL population in excel spreadsheet format (xls).** Each file contains angles for with both +UV-B (cellulose di-acetate) and -UV-B (Mylar). Click here for file

Additional File 10 **All measured cotyledon-opening angles, arranged by experimental round and RIL population in excel spreadsheet format (xls).** Each file contains angles for with both +UV-B (cellulose di-acetate) and -UV-B (Mylar). Click here for file

Additional File 11 **All measured cotyledon-opening angles, arranged by experimental round and RIL population in excel spreadsheet format (xls).** Each file contains angles for with both +UV-B (cellulose di-acetate) and -UV-B (Mylar). Click here for file

Additional File 12 **All measured cotyledon-opening angles, arranged by experimental round and RIL population in excel spreadsheet format (xls). **Each file contains angles for with both +UV-B (cellulose di-acetate) and -UV-B (Mylar). Click here for file

Additional File 13 **UV Spectra**. Additional description and a figure showing the spectral output of the UV313 bulbs and the irradiance under the Mylar-D and cellulose di-acetate filters used for -UV-B and +UV-B treatment of cotyledons. Click here for file
