# Supplementary material for: Chromosomal loci important for cotyledon opening under UV-B in Arabidopsis thaliana
Source: BMC Plant Biol. 2010 Jun 16;10:112. doi: 10.1186/1471-2229-10-112 (PMC3095277; doi:10.1186/1471-2229-10-112)
Supplement: Additional File 1 — Details about significant quantitative trait loci from the BayxSha mapping population. [file 1471-2229-10-112-S1.DOC]

**Additional Table 1 Bay x Sha Quantitative Trait Loci**

**A. Significant loci**

QTL variance components: Vg/Vp=0.1719 Ve/Vp=0.3079 Vge/Vp=0.0393 Vr/Vp=0.4809*

| **QTL** | **Chromosome** | **Marker Interval** | **QTL Position in cM** | **QTL Position Range in cM** | **Additive effect‡ ±SE (Pvalue)** | **h^2^ of QTLs** | **Confirmed in single-marker GLM** | **Round1** | | **Round2** | |
| --- | --- | --- | --- | --- | --- | --- | --- | --- | --- | --- | --- |
|  |  |  |  |  |  |  |  | **+UV-B** | **No UV-B** | **+UV-B** | **No UV-B** |
| BS1_64 | *1* | F5I14-MSAT1.13 | 64.7 | 60.3 - 68.7 | 9.26±1.49 (P<10^-6^) | 0.017 | F5I14 | NS | NS | NS | NS |
| BS2_25 | *2* | MSAT2.38-MSAT2.36 | 25 | 21 - 30.8 | -12.26±1.56 (P<10^-6^) | 0.036 | MSAT2.36 | NS | NS | -7.45±2.74 (P=0.0067)† | NS |
| BS3_6 | *3* | ATHCHIB2-MSAT3.19 | 6.8 | 4 - 11.8 | -9.43±1.43 (P<10^-6^) | 0.055 | ATCHIB2 | NS | NS | NS | NS |
| BS4_62 | *4* | MSAT4.9-MSAT4.37 | 62.9 | 56.9 – 68.9 | -7.18±1.78 (P=5.4x10^-5^) | 0.0085 | MSAT4.9 in round2 only | NS | NS | NS | NS |
| BS5_53 | *5* | MSAT5.9-MSAT5.12 | 53.2 | 43.4 – 57.2 | 13.68±1.67 (P<10^-6^) | 0.046 | MSAT5.9 | NS | NS | NS | NS |

NS= not significant.

*Vg is variance of genetic main effects, Vp is phenotypic variance, Ve is environmental (UV-B) effects, Vge is variance of genotype-by-environment interaction effects.

‡Positive numbers indicate that Bay allele is high, negative numbers indicate that Sha allele is high.

†Overall environmental heritability at this locus =0.0097.

**B. Significant epistasis between locus i and locus j**

| **QTL** | **Chr i** | **Markers i** | **Position i (in cM)** | **Range i (in cM)** | **Chr j** | **Markers j** | **Position j (in cM)** | **Range j** | **Additive epistatic effect SE (P value)** | **h^2^  of QTLs** | **UV-B specific epistasis** |
| --- | --- | --- | --- | --- | --- | --- | --- | --- | --- | --- | --- |
| BS2_25/5_53 | *2* | MSAT2.38-MSAT2.36 | 25 | 21 – 30.8 | *5* | MSAT5.9-MSAT5.12 | 53.2 | 43.4-57.2 | 5.78±1.82 (P=0.0015) | 0.0064 | NS |
