# Supplementary material for: Chromosomal loci important for cotyledon opening under UV-B in Arabidopsis thaliana
Source: BMC Plant Biol. 2010 Jun 16;10:112. doi: 10.1186/1471-2229-10-112 (PMC3095277; doi:10.1186/1471-2229-10-112)
Supplement: Additional File 2 — Details about significant quantitative trait loci from the ColxKas mapping population. [file 1471-2229-10-112-S2.DOC]

**Additional Table 2 Col x Kas Quantitative Trait Loci**

**A. Significant loci**

QTL Variance components: Vg/Vp=0.2177 Ve/Vp=0.0648 Vge/Vp=0.044 Vr/Vp=0.6735*

| **QTL** | **Chromosome** | **Marker Interval** | **QTL Position in cM** | **QTL Position Range in cM** | **Additive effect‡ ±SE (Pvalue)** | **h^2^** | **Confirmed in single-marker GLM** | **Round1** | | **Round2** | |
| --- | --- | --- | --- | --- | --- | --- | --- | --- | --- | --- | --- |
|  |  |  |  |  |  |  |  | **+UV-B** | **No UV-B** | **+UV-B** | **No UV-B** |
| CK2_75 | *2* | nga168-90J19T7 | 75.8 | 68.8-82.8 | 10.1±1.78 (P<10^-6^) | 0.059 | nga168 and 90J19T7 | NS | NS | NS | NS |
| CK4_27 | *4* | MSAT4.25-CD369(PCR) | 27.9 | 21.9-33.9 | -8.49±1.57 (P<10^-6^) | 0.045 | CD369(PCR) | -7.59±2.73 (P=0.0054)† | NS | NS | NS |
| CK5_26 | *5* | nga139-mi137 | 26.9 | 23- 34.9 | 5.47±1.46 (P=1.8x10^-4^) | 0.004 | nga139 | NS | NS | NS | NS |

NS= not significant.

*Vg is variance of genetic main effects, Vp is phenotypic variance, Ve is environmental (UV-B) effects, Vge is variance of genotype-by-environment interaction effects, Vr is residual variance.

‡positive numbers indicate that the Col allele is high, negative numbers indicate that Kas allele is high.

† overall environmental heritability at this locus =0.02

**B. Significant epistasis**

| **QTL** | **Chr i** | **Markers i** | **Position i (in cM)** | **Range i (in cM)** | **Chr j** | **Markers j** | **Position j (in cM)** | **Range j** | **Additive epistatic effect SE (P value)** | **h^2^** | **UV-B specific epistasis** |
| --- | --- | --- | --- | --- | --- | --- | --- | --- | --- | --- | --- |
| CK2_27/5_27 | 2 | nga168-90J19T7 | 75.8 | 68.8 – 82.8 | 5 | nga139-mi137 | 26.9 | 23- 34.9 | 7.96±1.86 (P=0.0015) | 0.0366 | NS |
| CK4_50/5_47 | 4 | nga1139-MSAT433 | 50.5 | 45.8-54.5 | 5 | MSAT522-CIW9 | 47.4 | 41.2-52.4 | -8.42±1.62 (P<10^-6^) | 0.0479 | NS |
