# Supplementary material for: Chromosomal loci important for cotyledon opening under UV-B in Arabidopsis thaliana
Source: BMC Plant Biol. 2010 Jun 16;10:112. doi: 10.1186/1471-2229-10-112 (PMC3095277; doi:10.1186/1471-2229-10-112)
Supplement: Additional File 3 — Details about significant quantitative trait loci from the LerxCol mapping population. [file 1471-2229-10-112-S3.DOC]

**Additional Table 3 Ler x Col Quantitative Trait Loci**

QTL variance components: Vg/Vp=0.3066 Ve/Vp=0.2741 Vge/Vp=0.0773 Vr/Vp=0.342*

| **QTL** | **Chromosome** | **Marker Interval** | **QTL Position in cM** | **QTL Position Range in cM** | **Additive effect‡ ±SE (Pvalue)** | **h^2^** | **Markers confirmed in single-marker GLM** | **Round1** | | **Round2** | |
| --- | --- | --- | --- | --- | --- | --- | --- | --- | --- | --- | --- |
|  |  |  |  |  |  |  |  | **+UV-B** | **No UV-B** | **+UV-B** | **No UV-B** |
| LCo2_41 | *2* | C2_067-C2_068 | 41 | 40-42 | -8.10±1.56 (P<10^-6^) | 0.039 |  | -14.01± 2.97 (P=2x10^-6^)† | NS | NS | 7.12±2.9 (P=0.017) |
| LCo4_30 | *4* | C4_042-C4_043 | 30 | 29-32 | 10.98±1.56 (P<10^-6^) | 0.07 | C4_042 and C4_043 | NS | NS | NS | NS |
| LCo5_9 | *5* | C5_017-C5_018 | 9 | 9-11 | -15.22±1.56 (P<10^-6^) | 0.151 | C4_017 and C5_018 | NS | -6.33  ±2.63 (P=0.016) | NS | NS |
| LCo5_61 | *5* | C5_105-C5_106 | 61 | 60-62 | 8.34±1.55 (P<10^-6^) | 0.039 | C5_105 and C5_106 | NS | NS | NS | NS |

NS= not significant.

*Vg is variance of genetic main effects, Vp is phenotypic variance, Ve is environmental (UV-B) effects, Vge is variance of genotype-by-environment interaction effects, Vr is residual variance.

‡positive numbers indicate that the Ler allele effect is higher, negative numbers indicate that Col allele is higher.

† overall environmental heritability at this locus =0.05
