# Supplementary material for: Chromosomal loci important for cotyledon opening under UV-B in Arabidopsis thaliana
Source: BMC Plant Biol. 2010 Jun 16;10:112. doi: 10.1186/1471-2229-10-112 (PMC3095277; doi:10.1186/1471-2229-10-112)
Supplement: Additional File 4 — Details about significant quantitative trait loci from the LerxCvi mapping population. [file 1471-2229-10-112-S4.DOC]

**Additional Table 4 Ler x Cvi**

**A. Significant loci**

Variance components: Vg/Vp=0.4294 Ve/Vp=01083. Vge/Vp=0.0221 Vr/Vp=0.4403*

| **QTL** | **Chromosome** | **Marker Interval** | **QTL Position in cM** | **QTL Position Range in cM** | **Additive effect‡ ±SE (Pvalue)** | **h^2^** | **Confirmed in single-marker GLM** |
| --- | --- | --- | --- | --- | --- | --- | --- |
| LCv1_8 | *1* | AXR1-HH.335C | 8 | 7-9 | -20.43±1.39 (P<10^-6^) | 0.162 | AXR1 and H.335C |
| LCv2_48 | *2* | ERECTA/GPA1-GD.298C | 48 | 47-51 | 18.37±1.39 (P<10^-6^) | 0.128 | ERECTA/GPA1 and GD.298C |
| LCv3_33 | *3* | AD.92L-GB.210L | 33 | 30-35 | -7.74±1.4 (P<10^-6^) | 0.014 | AD.92L |
| LCv4_63 | *4* | HH.159C-GB.490C | 63 | 61-65 | 15.55±1.45 (P<10^-6^) | 0.057 | HH.159C and GB.490C |
| LCv5_10 | *5* | BH.144L-EC.198L | 10 | 6-13 | 11.18±1.47 (P<10^-6^) | 0.026 | BH.144L and EC.198L |
| LCv5_104 | *5* | BF.168L-DF.119L | 104 | 99-109 | -6.16±1.34 (P=9x10^-6^) | 0.011 | BF.168L |

NS= not significant.

*Vg is variance of genetic main effects, Vp is phenotypic variance, Ve is environmental (UV-B) effects, Vge is variance of genotype-by-environment interaction effects, Vr is residual variance.

‡positive numbers indicate that the Ler allele is high, negative numbers indicate that Cvi allele is high.

**B. Significant epistasis**

| **QTL** | **Chr i** | **Markers i** | **Position i (in cM)** | **Range i (in cM)** | **Chr j** | **Markers j** | **Position j (in cM)** | **Range j** | **Additive epistatic effect SE (P value)** | **h^2^** | **UV-B specific epistasis** |
| --- | --- | --- | --- | --- | --- | --- | --- | --- | --- | --- | --- |
| LCv1_8/4_63 | *1* | AXR1-HH.335C | 8 | 7-9 | *4* | HH.159C-GB490C | 63 | 61-65 | -4.62±1.45 (P=0.0014) | 0.007 | NS |
