# Supplementary material for: Chromosomal loci important for cotyledon opening under UV-B in Arabidopsis thaliana
Source: BMC Plant Biol. 2010 Jun 16;10:112. doi: 10.1186/1471-2229-10-112 (PMC3095277; doi:10.1186/1471-2229-10-112)
Supplement: Additional File 13 — UV Spectra. Additional description and a figure showing the spectral output of the UV313 bulbs and the irradiance under the Mylar-D and cellulose di-acetate filters used for -UV-B and +UV-B treatment of cotyledons. [file 1471-2229-10-112-S13.DOC]

UV Spectra for Conte et al 2010 BMC Plant Biology

Spectral output (at cotyledon height) of UV313 bulbs covered with polyester (Mylar) and cellulose di-acetate (di-acetate), along with the output without a plastic filter for comparison. Irradiance was measured with a IL1700 double-monochromator spectroradiometer (International Light, Newburyport, MA), calibrated against a standard lamp (OL-40, Optronic, Orlando, FL) in the short-wavelength range and a model 1800 calibrator (LI-COR, Lincoln, NE) for wavelengths greater than 320 nm. Total UV-B in W m^-2^ was calculated by integrating the spectral irradiance between 290 and 315 nm.
